# Photodegradation and Biodegradation of Poly(Lactic) Acid Containing Orotic Acid as a Nucleation Agent

**DOI:** 10.3390/ma12030481

**Published:** 2019-02-04

**Authors:** Jan Salač, Jana Šerá, Martin Jurča, Vincent Verney, Adam A. Marek, Marek Koutný

**Affiliations:** 1 Department of Environmental Protection Engineering, Faculty of Technology, Tomas Bata University, Vavrečkova 275, 760 01 Zlín, Czech Republic; salac@utb.cz (J.S.); sera@utb.cz (J.Š.); m1_jurca@utb.cz (M.J.); 2 Institut de Chimie de Clermont-Ferrand, Université Clermont Auvergne, CNRS, F-63000 Clermont–Ferrand, France; vincent.verney@uca.fr; 3 Department of Organic Chemical Technology and Petrochemistry, Silesian University of Technology, 44100 Gliwice, Poland; adam.a.marek@polsl.pl

**Keywords:** poly(lactic acid), orotic acid, crystallinity, photodegradation, biodegradation, compost

## Abstract

Orotic acid is a natural heterocyclic compound that acts as a nucleation agent in poly(lactic acid) (PLA). PLA materials with increasing orotic acid content were prepared and characterized. It was found that crystallinity of about 28% was reached with 0.3% content of the agent. Further enhancement in the content of the agent did not provoke any additional significant increase of crystallinity. Subsequently, it was investigated whether the orotic acid content affected photodegradation of PLA and, in the next phase, its biodegradation. The results of rheological measurements showed that the compound slightly accelerates photodegradation of the material, which was accompanied by the cleavage of PLA chains. Previous photodegradation was shown to accelerate the subsequent biodegradation by shortening the lag phase of the process, where the explanation is probably in the reduction of the polymer molecular weight during the photodegradation. Moreover, the presence of orotic acid in both initial and photodegraded samples was found to influence biodegradation positively by shortening the lag phase and increasing the observed maximal rate of the biodegradation.

## 1. Introduction

More than 180 million tons of plastic waste are produced every year; a part of it enters the environment and causes problems due to its accumulation and omnipresence [1,2,3].

Currently, poly(lactide) acid (PLA) appears to be the most available biodegradable alternative to conventional polymers [4]. PLA is made through a combination of biotechnological and chemical processes, starting from renewable resources. PLA is biodegradable at higher temperatures only, approximately above 55 °C, which corresponds to biological composting processes [5]. On the other hand, PLA is not regarded as biodegradable in the soil environment [6]. PLA can find a number of applications, especially in the agricultural and food sectors [7,8].

The material properties of neat PLA are often not very favorable for some applications but they can be modified by various approaches known in polymer science [9]. One of the limiting features is a relatively high glass transition temperature of about 58 °C, making the material rather brittle at normal temperatures and the corresponding heat deflection temperature, which means that the PLA specimens tend to soften above a temperature of about 60 °C [10].

PLA materials that are processed in the form of melt in the conventional plastic processing technologies have usually very low crystallinity because the crystallization proceeds extremely slowly with crystal growth rate of less than 6 μm·min^−1^ [11]. For practical reasons, PLA materials usually cannot be annealed for a sufficiently long time during the production process. However, it was shown that a higher crystallinity could increase the elastic module up to about 25% and also considerably improve the heat deflection temperature [12,13]. The crystallinity of many polymer materials can be increased by the addition of nucleation agents, which provide initiation centres for the crystallization and also can increase the crystallization rate [14].

Various nucleation agents were examined with PLA, including some inorganic nucleating substances such as talc and clay [15,16,17,18] with some level of success. The general problem of the inorganic nucleation agents is their incompatibility with the polymer matrix and thus problems with even dispersion and prevention of their aggregation during the melt processing. Organic compounds with better solubility in the polymer matrix were also tested, including *N*,*N*-ethylene-bis(12-hydroxylstearamide) [19], and *N*,*N*′,*N*″-tricyclohexyl-1,3,5-benzene-tricarboxylamide (TMC-328) [20], where the latter was proven to be a very efficient nucleation agent, also able to improve the barrier properties of the material.

The ideal nucleation agent for PLA should also preserve its positive properties, especially biodegradability. Consequently, the compound must be nontoxic and biodegradable. Orotic acid (OA) appears to be a particularly good candidate. OA is a natural intermediate in the biosynthesis of pyrimidines [21,22,23] and was previously investigated and proved to be a relatively potent nucleation agent in PLA [24], though not so effective as some synthetic compounds e.g., *N*,*N*-(ethane-1,2-diyl)bis(*N*-phenoxylalamide) [25]. OA was also seen to improve the crystallization of polyhydroxy buthyrate [26].

This study aimed to verify the capacity of OA to act as a nucleation agent in PLA and especially to investigate whether the degradation of the resulting materials is altered. In particular, the photodegradation in controlled laboratory conditions and biodegradation during laboratory composting tests were studied. These processes are especially important for outdoor applications of PLA-based materials. It has been previously shown that crystallinity can affect the hydrolysis of ester bonds in PLA [5,27,28]; hence, in this case, some changes in the degradation processes could be expected. The influence of OA itself on the photodegradation, the biodegradation and the combination of both could be quite unpredictable and should be examined experimentally.

## 2. Materials and Methods

### 2.1. Preparation of Materials

**Figure 1:**
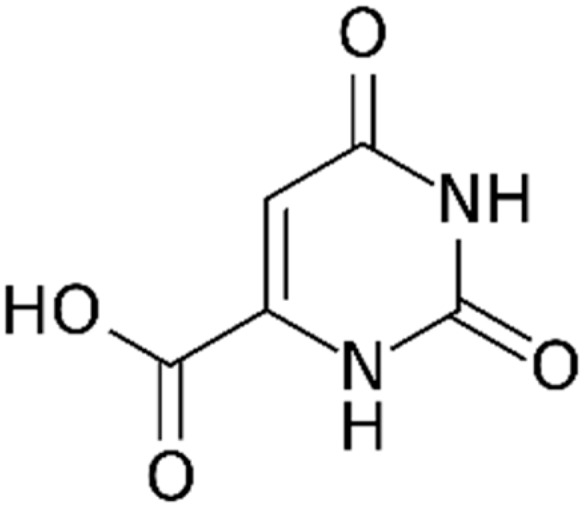
Orotic acid.

The polymer PLA2002D was obtained from NatureWorks^®^ Ingeo™ (Minnetonka, MN, USA); its weight average molecular weight (MW) was about 120 kg·mol^−1^. Orotic acid (Figure 1) was from Sigma-Aldrich (St. Louis, MO, USA). It was in the form of fine powder, the grains were of uneven shape, where the majority of particle sizes was in the interval from 7 to 30 µm. The distribution of the particle cross-section arreas was evaluated with optical microscopy using ImageJ 1.5 software (Wayne Rasband, National Institutes of Health, Bethesda, MD, USA) (Figure S1 in Supplementary Materials). OA has melting point of about 345 °C so it was still solid at the processing temperature. First, PLA masterbatch with 5% (*w*/*w*) of OA was prepared by melting and mixing the components in the two-screw laboratory mini-extruder DSM Xplore 15 (Xplore Instruments BV, Sittard, The Netherlands) at 190 °C for 5 min. Then materials containing 0.1, 0.3 and 0.5% (*w*/*w*) of OA were prepared under the same conditions by mixing the masterbatch and neat PLA. Finally, the materials were dried in the vacuum oven at 60 °C for 24 h. Sample films about 100 µm thick were prepared by compression molding at 180 °C and 20 MPa for 1 min. Then the hot plates were removed from the press and let to cool down slowly on air for about 2 h.

### 2.2. Scanning Electron and Optical Microscopy

#### 2.2.1. Scanning Electron Microscopy (SEM)

Particles of OA and PLA film materials were analyzed with the Phenom Pro (Thermo Fisher Scientific, Waltham, MA, USA) SEM. The samples were observed at the acceleration voltage of 10 kV in the backscattered electron mode. Measurements were carried out on samples without prior metallization using a special sample holder for Phenom Pro. Image analysis of the orotic acid particles was done with the ImageJ 1.5 software (Wayne Rasband, National Institutes of Health, Bethesda, MD, USA). The size of individual particles was determined from threshold-adjusted SEM images.

#### 2.2.2. Polarized Light Microscopy

PLA film materials were observed with polarized light microscopy ECLIPSE i50 (Nikon, Tokyo, Japan).

### 2.3. Differential Scanning Calorimetry (DSC)

For DSC measurement, Mettler Toledo DSC822 instrument (Mettler Toledo, Columbus, OH, USA) was used. The temperature range was set from 25 °C to 200 °C with the heating rate of 10 K·min^−1^ in air atmosphere. After heating, the samples were cooled down at the same rate and then, finally, the heating cycle was repeated. The degree of crystallinity was determined from the cooling trace with the help of the following equation:

(1) $$\chi_{c}=\left(\frac{\Delta H_{m}-\Delta H_{c}}{\Delta H_{m}^{0}\left(1-\frac{\%wt_{filler}}{100}\right)}\right)\times 100$$

where Δ*H_m_* is the heat of fusion, Δ*H_c_* is the cold crystallization enthalpy and $\Delta H_{m}^{0}$ is the tabulated theoretical heat of fusion for 100% crystalline PLA homopolymer (93.1 J·g^−1^) [29]. The measurements were done twice with identical results with the precision needed in this study (±1%).

### 2.4. Melt Rheology Study

Changes in the material on the molecular level were investigated by melt viscoelasticity experiments in oscillatory shearing mode using an advanced rheometry (ARES Rheometric Scientific T&A Instruments, New Castle, DE, USA) in the two parallel plate geometry (diameter 25 mm, gap between plates of 1 mm).

Intially, strain amplitude values were optimized for each sample to ensure that the measurements were done within the the linear viscoelastic region. Then, dynamic stresses were recorded at the optimal strain aplitude within the frequency interval from 0.1 to 100 rad·s^−1^ at 180 °C. The oscillatory frequency sweep test was applied to obtain resulting stress σ*(t) for an applied sinusoidal strain γ*(t). The complex dynamic viscosity η*(ω) can then be derived:

η*(ω) = σ*(t)γ*(t)
(2)

η*(ω) = η′(ω) − j η″(ω)
(3)

where η′(ω) is the loss viscosity and η″(ω) the storage viscosity and “j” is the complex number j^2^ = −1.

Data were plotted in the complex plane as so-called Cole-Cole graphs [30,31]. The relaxation in the entaglement polymer melt is manifested in the form of semi-circular traces of the measurement values during the oscillatory shearing. The Newtonian zero-shear viscosity η_0_, then corresponds to the intersection between the extrapolated semi-circle and the X-axis and is proportional to MW of the polymer:

η_0_ ∝ K∙(M_w_)^a^ (4)

where K and a are constants and a was determined to be in the interval 3.4–3.6 [32,33].

### 2.5. Photooxidation

Accelerated photo-ageing was performed in the ATLAS SEPAP 12/24 instrument (Atlas Material Testing Technology, Mount Prospect, IL, USA) [34] in air at 60 °C. The device is equipped with four 400 W mercury lamps (Mazda type MA 400) at each corner of the oven. UV-light radiation with wavelengths below 300 nm is filtered out by the glass envelopes of the sources. 2 cm × 2 cm film fragments were irradiated on aluminum holders on a rotating carousel. Films were exposed to the irradiation and temperature for up to 100 h.

### 2.6. Spectroscopic Characterization

Transmission UV-VIS spectra of the 100 µm PLA films were recorded in Shimadzu UV-2600 spectrometer (Shimadzu, Kyoto, Japan) in the wavelength interval from 200 nm to 800 nm. Fourier transform infrared (FTIR) spectra of the film samples in the transmission mode were obtained in Thermo Scientific Nicolet 6700 instrument (Thermo Fisher Scientific, Waltham, MA, USA).

### 2.7. Biodegradation Testing

Biodegradation tests [33] were realized in 500 mL flasks with septa mounted on the stoppers. The flasks contained polymer samples (50 mg), mature compost (5 g of dry weight, pH 7.1) and perlite (5 g). The flasks were incubated at 58 °C. Head space gas was sampled at appropriate intervals through the septum with a gas-tight syringe (100 μL), and injected into a gas chromatography (GC) instrument (Agilent 7890, Agilent Technologies, Santa Clara, CA, USA) equipped with Porapak Q (1.829 m length, 80/100 MESH) and 5A molecular sieve (1.829 m length, 60/80 MESH) columns connected in series and a thermal conductivity detector (carrier gas helium, flow 53 mL·min^−1^, column temperature 60 °C) to determine the concentration of CO_2_. The percentage of net mineralization with respect to the carbon content of the initial samples was calculated. Three parallel flasks were run for each sample, along with four blanks.

The experimental data were used to derive basic parameters of the biodegradation curves with the help of a mathematical model adapted from [35]:

(5) $$Cco_{2}=\frac{Cco_{2Max}}{1+\exp\left[2+\frac{4k}{Cco_{2Max}}\;\left(C-t\right)\right]}-\frac{Cco_{2Max}}{1+\exp\left[2+\frac{4k}{Cco_{2Max}}\;C\right]}$$

where $C_{CO_{2Max}}$ is the maximal level of evolved CO_2_ carbon expressed as the % of the maximal theoretical value; k represents the maximal rate of CO_2_ production (days^−1^) and C is the length of the lag phase (days). The modeling was done in open source software Gnuplot (version 5.2, Thomas Williams, Colin Kelley).

## 3. Results and Discussion

### 3.1. Preparation of Samples

PLA containing OA was prepared in a twin-screw extruder. First, a masterbatch containing 5% (*w*/*w*) of OA was made, then the individual samples with lower OA concentrations were prepared through the second extrusion process, including processing neat PLA reference material with zero concentration of OA. Thus, all samples finally had the identical processing history. Sample films were prepared by compression molding and then cooled slowly on air to facilitate the crystallization of the polymer.

### 3.2. Thermal Properties

**Figure 2:**
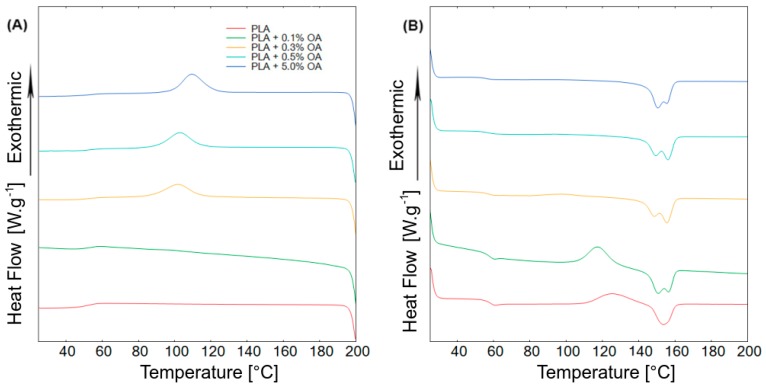
Differential scanning calorimetry. (**A**) Cooling traces; (**B**) heating traces with heat/cooling rate 10 K·min^−1^.

The basic thermal properties of the PLA samples with different OA contents and especially their tendency to crystallize were estimated in DSC experiments. The samples were first heated; here, the glass transition temperature and melting behavior was determined (Figure 2A, Table 1). Samples containing OA exhibited two distinct melting peaks. This phenomenon was already described [36] and explained by the presence of two types of crystals with identical α morphology but a different level of organization [37]. OA presence also caused a small positive shift in the glass transition temperature. During the cooling cycle, marked crystallization peaks were recorded in samples containing OA (Figure 2B). The crystallization temperature deduced was a bit higher than previously reported, which can be explained by a different type of PLA employed; however, the recorded enthalpy of crystallization appeared to be identical [24]. It was thus proven that OA acted as a nucleation agent. The degree of crystallization increased with the increasing content of OA, but it was evident that a further increase of the OA content above 0.3% did not lead to a marked increase in crystallinity (Table 1).

**Figure 3:**
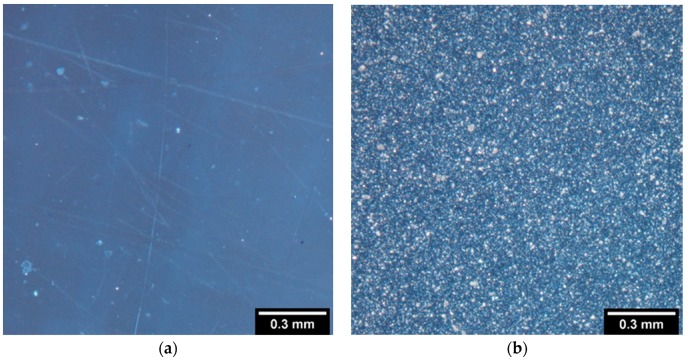
PLA films without OA (**a**) and with 0.3% of OA (**b**) as obtained by polarization microscopy.

The crystallization and uniform distribution of the nucleation agent was qualitatively confirmed by optical polarization microscopy (Figure 3). The important extent of the crystallization is clearly visible in the presence of OA; even distribution of the crystallites is also evident.

### 3.3. Photodegradation and Subsequent Analysis

**Figure 4:**
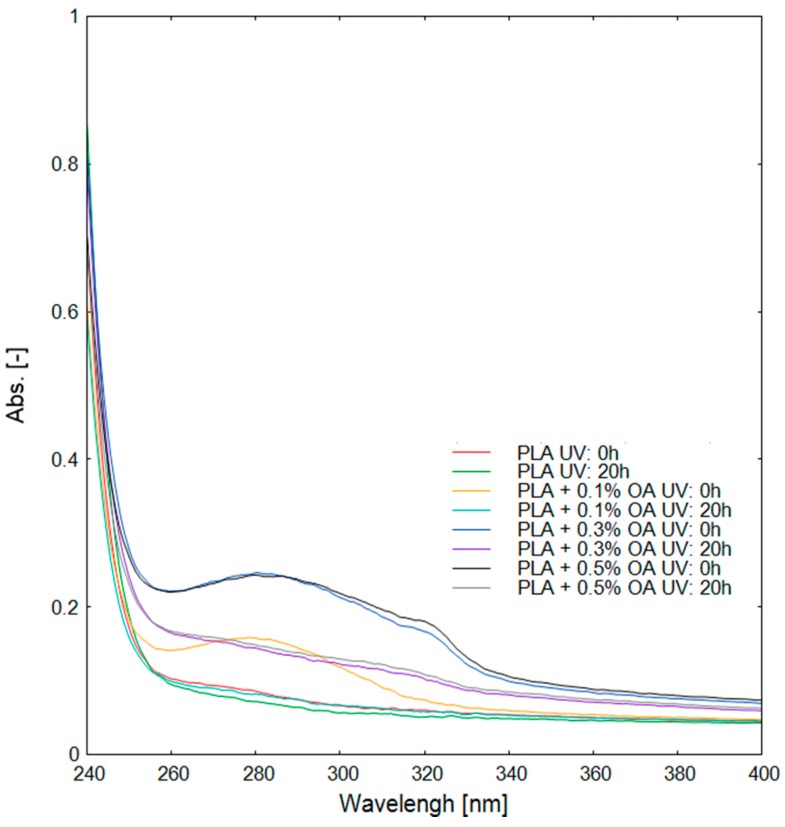
UV-VIS spectra of PLA materials containing different contents of OA before and after photodegradation.

The photodegradation of PLA-based materials by sun irradiation plays an important role, especially in agricultural applications. The photodegradation promotes changes in the materials that can influence their properties during its life cycle. To estimate such effects, a series of PLA materials with different OA contents (0.0%, 0.1%, 0.3%, and 0.5%) were subjected to the controlled artificial photodegradation experiment. Several increasing time periods of the exposure were chosen, where the maximal period of 100 hours should be equivalent to about three months of sun irradiation outdoors, during the summer season in the temperate climatic zone [38]. Changes in the material were evaluated with the help of FTIR and UV-VIS spectroscopy. It was suggested [39] that the photodegradation of PLA proceeds with the usual radical mechanism beginning with the abstraction of a tertiary hydrogen atom from a PLA chain with the formation of a radical, which then reacts with oxygen to form peroxide and subsequently hydroperoxide. The photolysis of the hydroperoxide then triggers the cleavage of the PLA chain. Only subtle changes were previously reported in FTIR spectra upon photodegradation of PLA [40]. Here, no evident spectral changes in the course of photodegradation could be discerned in FTIR spectra (Figure S2 in Supplementary Materials), even in the case of the maximal irradiation time (100 h), which was chosen to correspond to about one season of outdoor exposure and to be relevant to some agricultural applications. The surface of the samples was subjected to an inspection with SEM, but any structural changes or erosion were detected (Figure S3 in Supplementary Materials) after the photodegradation. On the other hand, the aromatic character of OA was revealed in UV-VIS spectra of the materials (Figure 4), where the aromatic component of the compound provoked a broad peak at about 280 nm. It could be seen that after 20 h of exposure, the OA peak was eliminated, providing an evidence of compound photodegradation.

**Figure 5:**
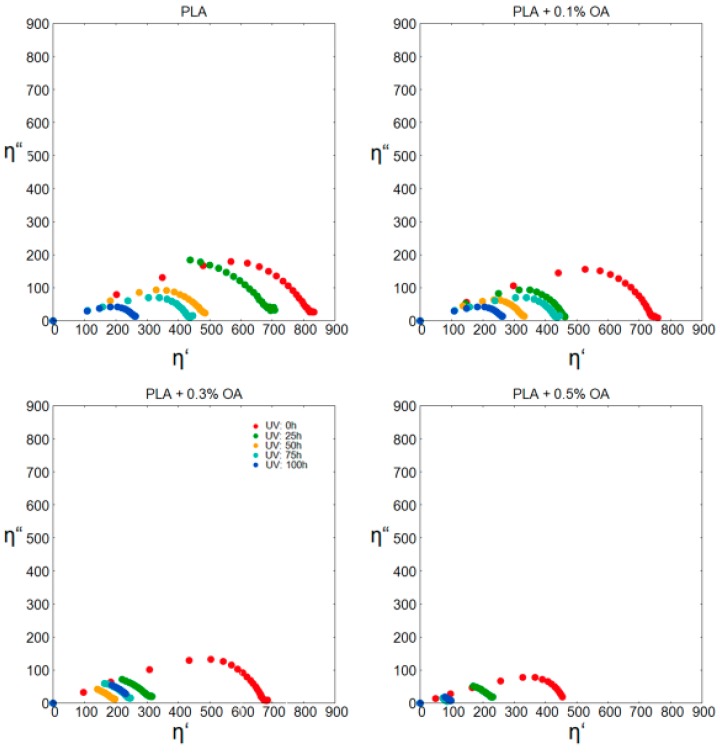
Cole-Cole plot for PLA materials with different contents of OA.

The photodegraded samples were further evaluated in melt rheology experiments using plate-plate geometry. Data for the PLA materials with the increasing content of OA were displayed in the form of Cole-Cole plots (Figure 5) as typical semicircle traces in the complex plane. It is evident that the storage modulus decreases with the increasing content of OA for non-irradiated samples; hence, OA provides some level of lubrication in the PLA matrix. The intersections of the semicircles with “x” axis correspond to the zero-shear viscosity (η_0_), which is proprotional to MW of the polymer (Equation (4)). Comparing the traces for the increasing irradiation times, it is obvious that the photodegradation was accompanied by polymer chain scissions and resulted in a marked decrease in its MW.

**Figure 6:**
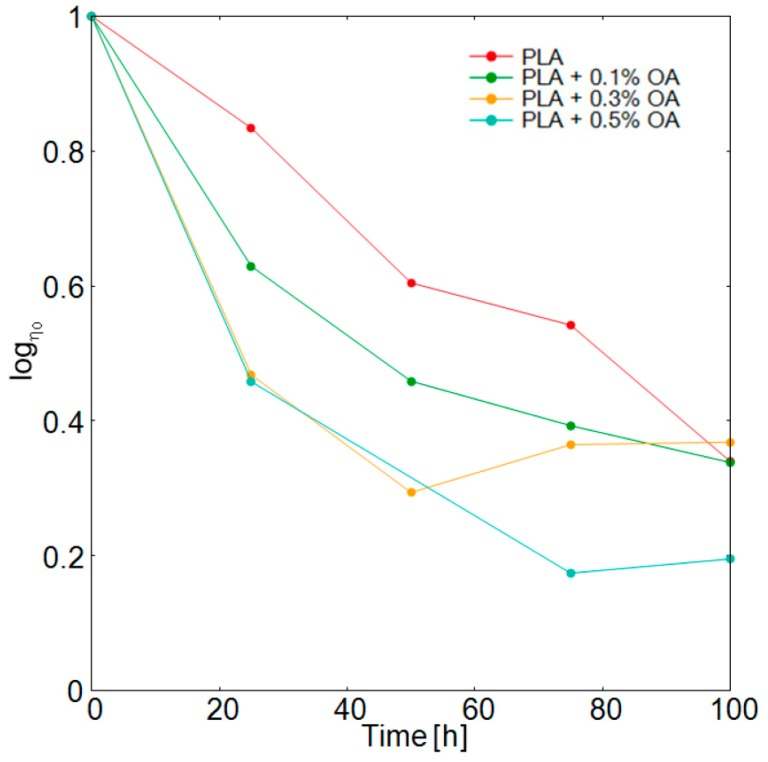
Dependence of the zero shear viscosity of PLA materials with different contents of OA on the period of photodegradation.

Deriving the zero shear viscosities and plotting them against the period of photooxidation (Figure 6), the kinetic of the zero shear viscosity decrease and the corresponding decrease in MW of the polymer is much better discernable. In the initial phase, the rate of the zero shear viscosity decrease corresponded well with the content of OA, with the gradual increase of the initial rate up to AO content of 0.3%. Thus, in the early phase of the photodegradation, OA acted as a photosensitizer and promoted a higher rate of chain scissions. In consequence, it can be seen that the rate of the zero shear viscosity decrease and so the corresponding decrease of MW was proportional to the content of OA. Employing Equation (4) and the published values of the constants [30,31], it could be estimated that MW of the material with 0.3% OA content dropped to about 80 kg·mol^−1^ at the end of the period observed in Figure 5. In the later phase of the photodegradation, OA was probably degraded already, as could be assumed from UV-VIS spectra (Figure 4), and the zero shear viscosity for all materials with different contents of OA leveled at about 0.3 Pa·s^−1^ (Figure 6).

### 3.4. Biodegradation in Compost

Selected samples of PLA materials both before and after photodegradation were subjected to biodegradation in the compost environment at 58 °C. The material with 0.3% OA content was selected because it shows high crystallinity at a reasonable content of the additive and thus is the most relevant for a real application. The aim of the experiment was to investigate the influence of the OA presence, and eventually of the increased crystallinity induced by OA during the biodegradation. Furthermore, the experiment should also show the impact of the previous photodegradation on the biodegradation. It is well accepted [41] that the biodegradation of PLA is preceded by the chemical hydrolysis of ester bonds in the polymer, which is connected with the decrease of MW to the level accessible by the enzymatic systems of microorganisms [26]. Hence, the photodegradation, which was proven to decrease MW, can support somewhat faster biodegradation.

**Figure 7:**
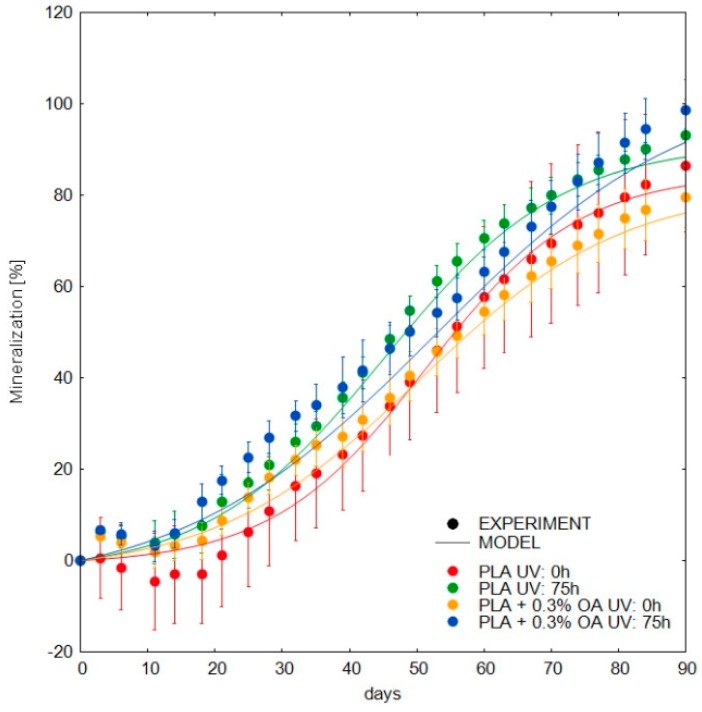
Biodegradation of PLA materials in compost at 58 °C.

There were no significant differences between the biodegradation curves of photodegraded and initial PLA materials (Figure 7). All samples reached almost full mineralization, i.e., metabolization of carbon in the material into carbon dioxide, in about 90 days. Judging just by sight, non-irradiated samples exhibited longer lag phases on the beginning of the process. After a more detailed comparison and the deduction of the kinetic parameters of the biodegradation curves, it was found that photodegraded samples exhibited faster mineralization (Table 2). It was especially evident that the lag phases were shorter for the photodegraded samples, which is consistent with the drop in MW, demonstrated in rheological measurements. As already mentioned, a lag phase is a typical feature of PLA biodegradation, during which relative molecular weight has to decrease to about 30,000, driven by the abiotic hydrolysis, before the CO_2_ evolution from the biological processes can be observed [41]. MW reduction during the photodegradation thus tend to shorten the lag phase during the biodegradation. On the other hand, as seen from the rheological measurements and UV-VIS and FTIR spectra, the photosensitization caused by the OA presence seemed to be very weak and transient.

The maximal rate of the subsequent CO_2_ production appeared to be almost identical between the initial and photodegraded samples, which suggested that the photodegradation did not influence the process through other factors, e.g., the formation of inhibiting compounds.

The comparison of the corresponding samples with and without OA is interesting. Both lag phases and maximal rates suggested that OA presence supported faster biodegradation. In some previous studies, it was outlined that crystallinity itself did not retard biodegradation [27,28]. Moreover, as shown previously [33], during the initial lag phase, the cold crystallization induced by chain scissions tended to increase the crystallinity considerably, up to about 30% and thus probably the crystallinities in all samples were equalized at the end of the lag phase. Another mechanism then should be sought to explain the acceleration of the biodegradation, e.g., catalysis of the ester bond hydrolysis in PLA by OA or by degradation products of OA formed by the photodegradation.

## 4. Conclusions

With growing pressure to restrict conventional plastic, at least in some applications, there is the growing need to seek alternatives for materials in various biodegradable plastics. A very challenging task is then the effort to improve the properties of the latter materials to meet the demands for particular applications and at the same time preserve wanted properties e.g., above all, biodegradability.

In the study, it has been proven that OA can be employed as a relatively potent crystallization agent in PLA, where the improved crystallinity could be very important for certain applications. Although OA can effectively absorb UV irradiation, it was not identified to be a strong photosensitization agent in given concentrations, and degraded rather rapidly by the action of UV irradiation in the employed concentrations. The photodegradation, regardless of the OA presence, shortened the lag phase of the PLA biodegradation, where the mechanism lay most probably in the reduction of MW during the photodegradation.

Moreover, it was shown that OA presence did not negatively interfere with the biodegradability of PLA, which is the crucial and most valuable property of this material. OA itself or the crystallinity induced by the OA presence did not retard the biodegradation; even so, the presence of OA seemed to shorten the lag phase and increase the observed maximal rates of the biodegradation.

## Figures and Tables

**Table 1:** DSC-derived parameters for PLA with the OA content.

Material	*T*_g_ (°C)	*T*_m1_ (°C)	*T*_m2_ (°C)	*T*_c_ (°C)	*H*_g_ (J·g^−1^·K^−1^)	*H*_c_ (J·g^−1^)	*χ*_c_ (%)
PLA	57	153	nd	nd	0.53	0	0
PLA + 0.1% OA	58	150	156	99	0.40	1.8	2
PLA + 0.3% OA	59	148	155	102	0.29	26.2	28
PLA + 0.5% OA	59	149	156	103	0.26	28.6	31
PLA + 5.0% OA	59	150	155	110	0.27	31.8	34

*T*_g_, glass transition temperature; *T*_m1_ and *T*_m2_, melting temperature; *T*_c_, temperature of crystallization; *H*_g_, enthalpy of glass transition; *H*_c_, enthalpy of crystallization; *χ*_c_, degree of crystallinity.

**Table 2:** Kinetics parameters of the biodegradation obtained from Equation (5).

Parameter	PLA		PLA + 0.3% OA	
UV (h)	0	75	0	75
CO_2_Max_^a^ (%)	86 ± 2.5	95 ± 1.1	85 ± 2.1	112 ± 5.1
k ^b^ (day^−1^)	1.9 ± 0.09	1.8 ± 0.04	1.4 ± 0.04	1.5 ± 0.05
C ^c^, (days)	28.4 ± 1.2	18.5 ± 0.57	19.9 ± 0.92	14.7 ± 1.4
R^2^	0.991	0.994	0.996	0.991

^a^ Maximal level of mineralization; ^b^ maximal rate of mineralization; ^c^ duration of the lag phase, values ± standard deviations.

## Supplementary Materials

The following are available online at http://www.mdpi.com/1996-1944/12/3/481/s1, Figure S1: (a) SEM micrographs of OA powder, (b) Threshold sharpened images of OA grains used for the image analysis, (c) Distribution of cross-section areas of OA grains. Figure S2: FTIR spectra of the investigated PLA materials with different contents of OA, both before and after 20 h of the photodegradation. Figure S3: SEM of PLA materials before and after photodegradation. PLA (a), PLA after 75 h of photodegradation (b), PLA with 0.3% of OA (c), PLA with 0.3% of OA after 75 hours of photodegradation (d).
