# Supplementary material for: Photodegradation and Biodegradation of Poly(Lactic) Acid Containing Orotic Acid as a Nucleation Agent
Source: Materials (Basel). 2019 Feb 4;12(3):481. doi: 10.3390/ma12030481 (PMC6384750; doi:10.3390/ma12030481)
Supplement: Supplementary file 1 [file materials-12-00481-s001.pdf]

Photodegradation and Biodegradation of Poly(Lactic) Acid Containing Orotic Acid as a Nucleation Agent

Jan Salač [1], Jana Šerá [1], Martin Jurča [1], Vincent Verney [2], Adam Marek [3] and Marek Koutný [1,*]

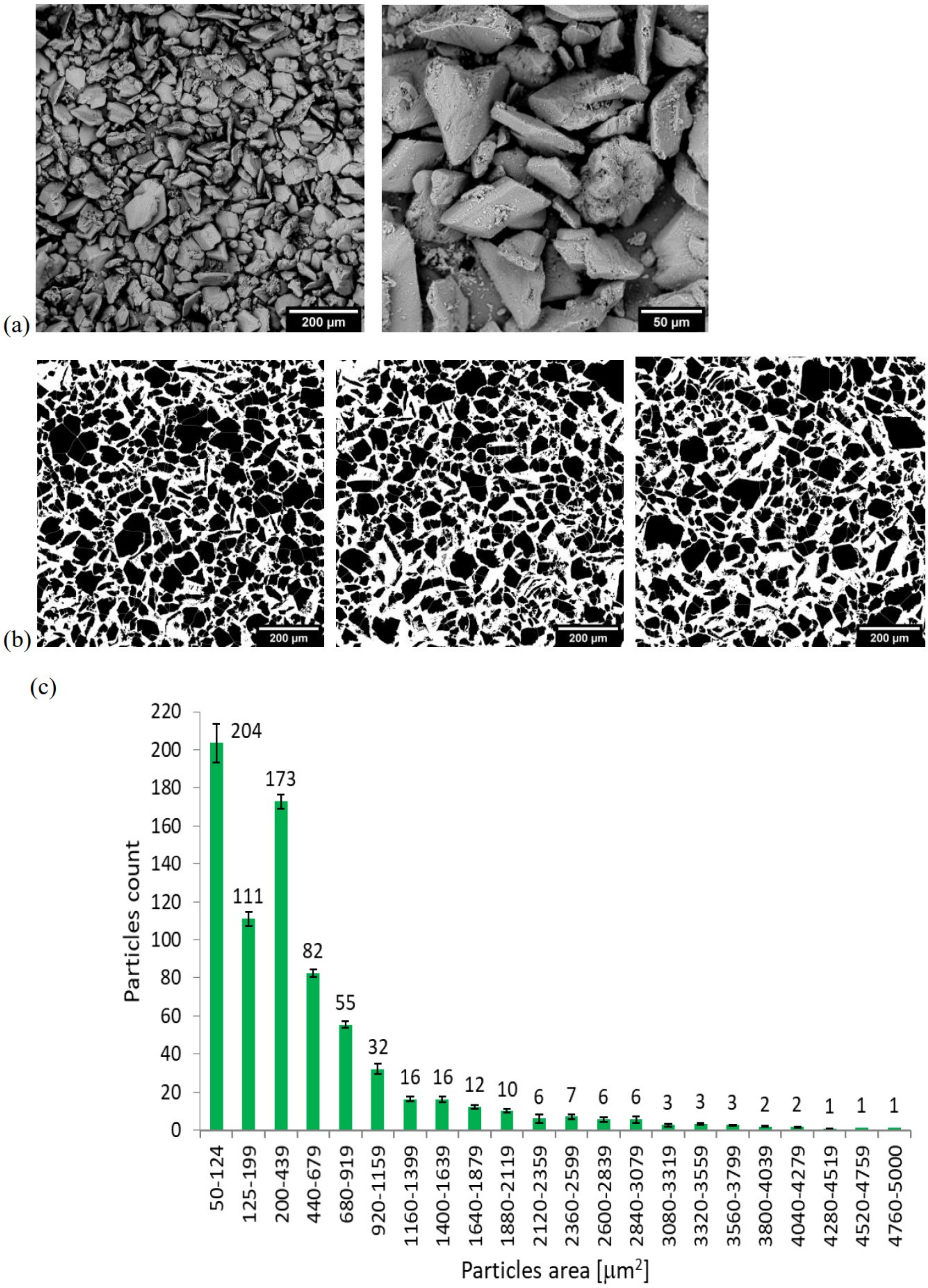

Figure S1. (**a**) SEM micrographs of OA powder. (**b**) Threshold sharpened images of OA grains used for the image analysis. (**c**) Distribution of cross-section areas of OA grains.

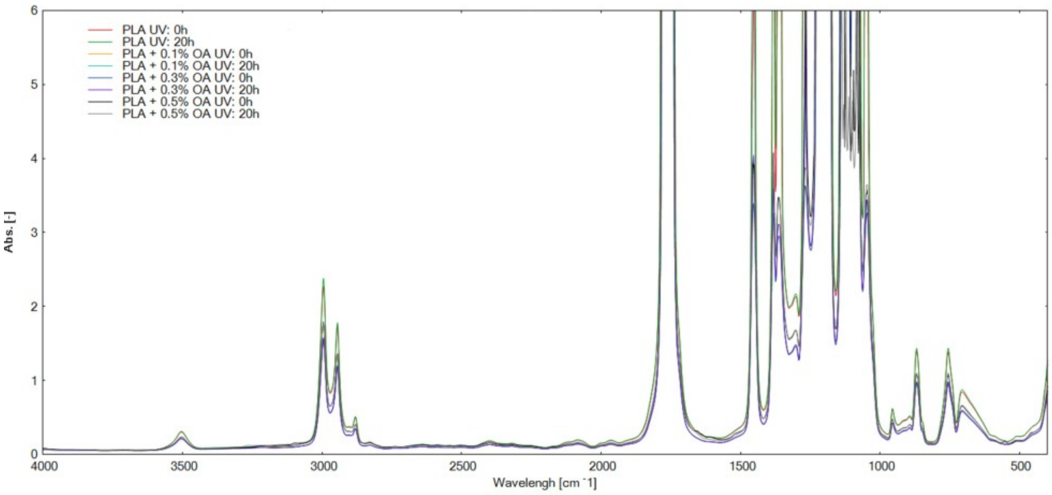

Figure S2. FTIR spectra of the investigated PLA materials with different contents of OA, both the initial and after 20 h of the photodegradation.

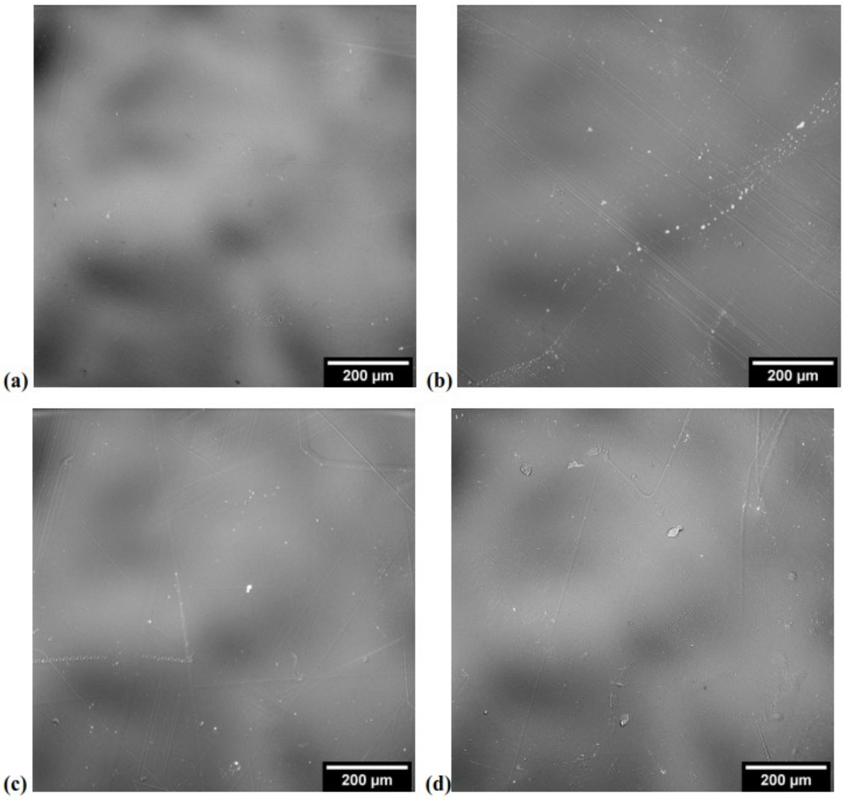

Figure S3. SEM of PLA materials before and after photodegradation. PLA (**a**), PLA after 75 hours of photodegradation (**b**), PLA with 0.3% of OA (**c**), PLA with 0.3% of OA after 75 h of photodegradation (**d**).
